# On the accuracy of one- and two-particle solvation entropies

**DOI:** 10.1063/1.4983654

**Published:** 2017-05-19

**Authors:** Benedict W. J. Irwin, David J. Huggins

**Affiliations:** 1 Theory of Condensed Matter Group, Cavendish Laboratory, University of Cambridge, 19 J J Thomson Avenue, Cambridge CB3 0HE, United Kingdom; 2 Department of Chemistry, University of Cambridge, Lensfield Road, Cambridge CB2 1EW, United Kingdom

## Abstract

Evaluating solvation entropies directly and combining with direct energy calculations is one way of calculating free energies of solvation and is used by Inhomogeneous Fluid Solvation Theory (IFST). The configurational entropy of a fluid is a function of the interatomic correlations and can thus be expressed in terms of correlation functions. The entropies in this work are directly calculated from a truncated series of integrals over these correlation functions. Many studies truncate all terms higher than the solvent-solute correlations. This study includes an additional solvent-solvent correlation term and assesses the associated free energy when IFST is applied to a fixed Lennard-Jones particle solvated in neon. The strength of the central potential is varied to imitate larger solutes. Average free energy estimates with both levels of IFST are able to reproduce the estimate made using the Free energy Perturbation (FEP) to within 0.16 kcal/mol. We find that the signal from the solvent-solvent correlations is very weak. Our conclusion is that for monatomic fluids simulated by pairwise classical potentials the correction term is relatively small in magnitude. This study shows it is possible to reproduce the free energy from a path based method like FEP, by only considering the endpoints of the path. This method can be directly applied to more complex solutes which break the spherical symmetry of this study.

## I. INTRODUCTION

The ability to estimate the free energy difference between two defined states is a useful tool in computational chemistry. Direct applications are predicting the free energy of a solvation process, whether it may be testing a small molecule in a solvent to see if the pair is likely to be miscible^1^ or a larger system, for example, a peptide, protein,^2^ or protein-ligand complex.^3^ The latter has a direct implication to *in silico* drug designs. Being able to quantitatively measure such a change then allows the relative comparison of ligands for a given protein.^4^ For the protein-ligand complex, if the free energy of the bound state is less than the unbound state then the equilibrium will favour the bound state.

There exist a number of methods of estimating changes in the free energy with computational simulations. These include the Free Energy Perturbation (FEP)^5^ and Thermodynamic Integration (TI).^6^ Both these methods rely on a well defined path from the reference state to the new state, but are very generally applicable and work with different levels of theory for the description of the physical system.^7^ Another method which has seen growing success is Inhomogeneous Fluid Solvation Theory (IFST),^8–10^ which does not need a well-defined path between the reference state and the new state; however, IFST can only calculate changes in the free energy in the context of a solvation process. IFST performs this by using a direct computation of the change in entropy due to solvation and combining this with a direct energy measurement^11,12^ to calculate the free energy. There are numerous examples of calculating and estimating such a change in free energy.^13–18^

This work attempts to expand on one method of measuring the solvation entropy change directly, namely the Mutual Information Expansion (MIE),^19^ and uses a k-Nearest Neighbours (KNN)^20,21^ estimator to evaluate an approximation to the change in the solvation entropy. This work is different from the majority of previous works, in that we truncate the MIE at a higher order, including an additional term. This additional term represents correlations between two solvent molecules in the presence of a solute, and such terms have been measured before in the context of water in protein binding pockets^22^ using Grid Inhomogeneous Solvation Theory (GIST).^23,24^ We seek to find quantitatively, the change in free energy associated with the extra information, and whether it is necessary to include this term in the MIE. The system under study in this work is fundamental and simple, a Lennard-Jones neon solvent with a fixed Lennard-Jones atom in the centre of the simulation cell which represents the solute in a solvation process. The changes in free energy will be compared to an equivalent FEP simulation. The comparison of FEP and IFST in this work is independent of the forcefield used.

First, we will discuss the theory used in all calculations, then review the computational methods used, and input simulation parameters. The results will then be discussed and then analysed.

## II. THEORY

**FIG. 1.**
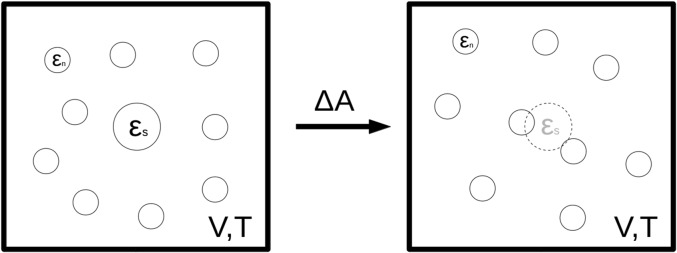
Schematic of the canonical ensemble simulation for both IFST and FEP. The *N* solvent neons with the Lennard-Jones parameter $\varepsilon_{n}$ surround the solute with the Lennard-Jones parameter $\varepsilon_{s}$ (left). The solute interactions are turned off, while the volume and temperature remain constant (right). Both methods used in this study are measuring the change in the Helmholtz free energy, ΔA, between these two states.

Our calculations of the solvation free energy associated with fixing a Lennard-Jones atom at the centre of a neon simulation box (see Fig. 1) are based on the following:

*Step 1*. A change in free energy in the canonical ensemble is given by a change in the Helmholtz free energy,

ΔA=ΔU−TΔS, (1)

where ΔU is the change in the internal energy of the system, *T* is the temperature of the system, and ΔS is the change in the entropy of the system. The changes here are the differences between the solute-liquid system and the bulk liquid system.

*Step 2*. By using a molecular dynamics (MD) simulation with a parameterized forcefield, it is possible to estimate ΔU as

$$\Delta U={\bar{U}}_{2}-{\bar{U}}_{1},$$ (2)

where ${\bar{U}}_{1}$ is the equilibrium expectation energy of a system of *N* neon atoms in a periodic cell of volume *V* at temperature *T*, and ${\bar{U}}_{2}$ is the equilibrium expectation energy of a system of *N* neon atoms in the presence of a fixed Lennard-Jones atom at volume *V* and temperature *T*.

*Step 3*. It is possible to write the total change in solvation entropy, ΔS in Eq. (1), as an expansion over correlations of one-body, two-body, three-body, and so on, as discussed by Baranyai and Evans^25^ for the homogeneous fluid and by Lazaridis^8,9^ for the inhomogeneous fluid. The theory for homogeneous fluids was extensively studied by Kirkwood,^6,26^ Nettleton,^27^ Raveche,^28^ and Wallace.^29–31^

For the system of *N* ideal atoms with coordinates ${\left\{𝐫\right\}}_{N}=\left\{{𝐫}_{1},\ldots ,{𝐫}_{N}\right\}$ and momenta ${\left\{𝐩\right\}}_{N}=\left\{{𝐩}_{1},\ldots ,{𝐩}_{N}\right\}$, we have the *N* body distribution in positions and momenta

$$f_{N}=f_{N}\left({\left\{𝐫\right\}}_{N},{\left\{𝐩\right\}}_{N}\right)=g_{N}\left({\left\{𝐫\right\}}_{N}\right)\prod_{k=1}^{N}f_{k}\left({𝐩}_{k}\right),$$ (3)

and the total entropy of a bulk fluid is given by

$$S_{\text{liquid}}=-\frac{Rh^{3N}}{N!}\int f_{N}\ln f_{N}d{\left\{𝐫\right\}}_{N}d{\left\{𝐩\right\}}_{N},$$ (4)

with *R* the gas constant and *h* the Planck constant. Then, the separability of the momentum can be exploited to give

$$S_{\text{liquid}}=S_{\text{momentum}}+S_{\text{configuration}},$$ (5)

which is written explicitly as

$$S_{\text{liquid}}=\underset{\text{Momentum}}{\underbrace{-\frac{NR}{\rho }\int f_{1}\left({𝐩}_{1}\right)\ln f_{1}\left({𝐩}_{1}\right)d{𝐩}_{1}}}\underset{\text{Configuration}}{\underbrace{-\frac{R\rho^{N}}{N!}\int g_{N}\left({\left\{𝐫\right\}}_{N}\right)\ln g_{N}\left({\left\{𝐫\right\}}_{N}\right)d{\left\{𝐫\right\}}_{N}}}.$$ (6)

The momentum terms are the same as those of an ideal gas where the one-body distribution of momenta is given by

$$f_{1}\left(𝐩\right)=\rho {\left(2\pi mkT\right)}^{-3/2}\exp\left(\frac{{𝐩}^{2}}{2mkT}\right),$$ (7)

where *k* is the Boltzmann constant, *ρ* is the number density of the equivalent ideal gas, and *m* is the mass of the atom. Then,

$$\begin{array}{cc} S_{\text{momentum}} & =-\frac{NR}{\rho }\int f_{1}\left({𝐩}_{1}\right)\ln f_{1}\left({𝐩}_{1}\right)d{𝐩}_{1}\\ & =\frac{3NR}{2}-NR\ln\left(\rho \lambda^{3}\right), \end{array}$$ (8)

with *λ* the thermal wavelength of an atom in the liquid.

For a vector of random variables $𝐗=\left(X_{1},\ldots ,X_{n}\right)$, we may write^32^

$$H\left(𝐗\right)=H\left(X_{1}\right)+H\left(X_{2}|X_{1}\right)+H\left(X_{3}|X_{1},X_{2}\right)+\cdots +H\left(X_{n}|X_{1},\cdots ,X_{n-1}\right),$$ (9)

where *H* is an information entropy and the notation *H*(*X*|*Y*) is the conditional entropy of *X* given *Y*. In a similar fashion, we may write

$$\underset{\text{Configuration}}{\underbrace{-\frac{R\rho^{N}}{N!}\int g_{N}\left({\left\{𝐫\right\}}_{N}\right)\ln g_{N}\left({\left\{𝐫\right\}}_{N}\right)d{\left\{𝐫\right\}}_{N}}}=S_{2}-I_{3}+I_{4}-\cdots$$ (10)

giving

$$S_{\text{liquid}}=S_{\text{momentum}}+S_{2\text{ liquid}}-I_{3\text{ liquid}}+I_{4\text{ liquid}}-\cdots$$ (11)

This expansion was performed for a homogeneous fluid by Wallace. It applies to the canonical ensemble and is non-local, meaning that it is only exact when integrated over the entire system volume.^25,29^ An ensemble invariant and a local form of the expansion are discussed by Baranyai and Evans.^25^ In the conditions of this study, the distinguishing terms between the local and non-local forms cancel, so here the non-local form is used for simplicity. In this case, the expansion has the following terms:

$$S_{\text{momentum}}=-\frac{NR}{\rho }\int f_{N}^{\left(1\right)}\left(𝐩\right)\ln h^{3}f_{N}^{\left(1\right)}\left(𝐩\right)d𝐩,$$ (12)

$$S_{2\text{ liquid}}=-\frac{R\rho^{2}}{2!}\int \int g_{N}^{\left(2\right)}\left({𝐫}_{1},{𝐫}_{2}\right)\ln g_{N}^{\left(2\right)}\left({𝐫}_{1},{𝐫}_{2}\right)d{𝐫}_{1}d{𝐫}_{2},$$ (13)

$$I_{3\text{ liquid}}=\frac{R\rho^{3}}{3!}\int \int \int g_{N}^{\left(3\right)}\left({𝐫}_{1},{𝐫}_{2},{𝐫}_{3}\right)\ln\delta g_{N}^{\left(3\right)}\times \left({𝐫}_{1},{𝐫}_{2},{𝐫}_{3}\right)d{𝐫}_{1}d{𝐫}_{2}d{𝐫}_{3}.$$ (14)

We can write the part of the three-body correlation function which cannot be expressed multiplicatively by its marginal distributions as

$$\delta g_{N}^{\left(3\right)}\left({𝐫}_{1},{𝐫}_{2},{𝐫}_{3}\right)=\frac{g_{N}^{\left(3\right)}\left({𝐫}_{1},{𝐫}_{2},{𝐫}_{3}\right)}{g_{N}^{\left(2\right)}\left({𝐫}_{1},{𝐫}_{2}\right)g_{N}^{\left(2\right)}\left({𝐫}_{1},{𝐫}_{3}\right)g_{N}^{\left(2\right)}\left({𝐫}_{2},{𝐫}_{3}\right)},$$ (15)

where each of these *N* particle correlation functions for *k* bodies can be written in terms of the *k* body density as^33^

$$g_{N}\left({𝐫}_{1},{𝐫}_{2},\ldots ,{𝐫}_{k}\right)=\frac{1}{\rho^{k}}\rho_{N}\left({𝐫}_{1},{𝐫}_{2},\ldots ,{𝐫}_{k}\right).$$ (16)

Finally, we may write the excess entropy of the liquid as

$$S_{\text{excess liquid}}=S_{\text{liquid}}-S_{\text{ideal}},$$ (17)

we have

Sideal=Smomentum+R=5NR2−NR ln(ρλ3). (18)

Therefore,

$$S_{\text{excess liquid}}=S_{2\text{ liquid}}-I_{3\text{ liquid}}+I_{4\text{ liquid}}-\cdots -R,$$ (19)

where terms with an *S* denote an entropy and terms with an *I* denote a mutual information term. The expansion with these terms is true for a homogeneous fluid. For the simulations used in this work, we include the presence of the central solute.^8^ The central solute in our calculations is spherically symmetric, and our end goal is to generalise to any solute and solvent, so we must consider an inhomogeneous system. This was analytically performed by Lazaridis.^8,9^

Eqs. (12)–(14) are the relevant terms for the liquid, and for a system with a solute we may write

$$S_{1\text{ solute}}=-R\rho \int g_{N}^{\left(1\right)}\left({𝐫}_{1}|s\right)\ln g_{N}^{\left(1\right)}\left({𝐫}_{1}|s\right)d{𝐫}_{1},$$ (20)

$$I_{2\text{ solute}}=\frac{R\rho^{2}}{2!}\int \int g_{N}^{\left(2\right)}\left({𝐫}_{1},{𝐫}_{2}|s\right)\ln\delta g_{N}^{\left(2\right)}\left({𝐫}_{1},{𝐫}_{2}|s\right)d{𝐫}_{1}d{𝐫}_{2},$$ (21)

$$I_{3\text{ solute}}=-\frac{R\rho^{3}}{3!}\int \int \int g_{N}^{\left(3\right)}\left({𝐫}_{1},{𝐫}_{2},{𝐫}_{3}|s\right)\ln\delta g_{N}^{\left(3\right)}\times \left({𝐫}_{1},{𝐫}_{2},{𝐫}_{3}|s\right)d{𝐫}_{1}d{𝐫}_{2}d{𝐫}_{3},$$ (22)

where the |*s* argument indicates the presence of a solute. We can write the part of the two- and three-body correlation functions which cannot be expressed by its marginal distributions as

$$\delta g_{N}^{\left(2\right)}\left({𝐫}_{1},{𝐫}_{2}|s\right)=\frac{g_{N}^{\left(2\right)}\left({𝐫}_{1},{𝐫}_{2}|s\right)}{g_{N}^{\left(1\right)}\left({𝐫}_{1}|s\right)g_{N}^{\left(1\right)}\left({𝐫}_{2}|s\right)},$$ (23)

$$\delta g_{N}^{\left(3\right)}\left({𝐫}_{1},{𝐫}_{2},{𝐫}_{3}|s\right)=\frac{g_{N}^{\left(3\right)}\left({𝐫}_{1},{𝐫}_{2},{𝐫}_{3}|s\right)g_{N}^{\left(1\right)}\left({𝐫}_{1}|s\right)g_{N}^{\left(1\right)}\left({𝐫}_{2}|s\right)g_{N}^{\left(1\right)}\left({𝐫}_{3}|s\right)}{g_{N}^{\left(2\right)}\left({𝐫}_{1},{𝐫}_{2}|s\right)g_{N}^{\left(2\right)}\left({𝐫}_{1},{𝐫}_{3}|s\right)g_{N}^{\left(2\right)}\left({𝐫}_{2},{𝐫}_{3}|s\right)}.$$ (24)

Some slight differences exist between the homogeneous and inhomogeneous formulations, $S_{2\text{ liquid}}$ is not a mutual information term (denoted with an *I* rather than an *S*), as the marginal distributions vanish. We may then write

$$S_{\text{ solute}}=S_{\text{momentum}}+S_{1\text{ solute}}-I_{2\text{ solute}}+I_{3\text{ solute}}-\cdots$$ (25)

and subtracting the ideal gas terms, Eq. (18),

$$S_{\text{excess solute}}=S_{1\text{ solute}}-I_{2\text{ solute}}+I_{3\text{ solute}}-\cdots -R.$$ (26)

To find the change in entropy from the addition of the solute, we calculate the *excess entropy of solution*, $\Delta S_{\text{exc soln}}$. This is then the excess entropy of the solute system, Eq. (26), minus the excess entropy of the neat liquid, Eq. (19),

$$\Delta S_{\text{exc soln}}=S_{\text{excess solute}}-S_{\text{excess liquid}},$$ (27)

$$\Delta S_{\text{exc soln}}=\left[S_{1\text{ solute}}-I_{2\text{ solute}}+I_{3\text{ solute}}-\cdots \right]-\left[S_{2\text{ liquid}}-I_{3\text{ liquid}}+I_{4\text{ liquid}}-\cdots \right],$$ (28)

where we see the factors of −*R* from $S_{\text{excess solute}}$ and $S_{\text{excess liquid}}$ cancel. It is at this point we choose a truncation of $\Delta S_{\text{exc soln}}$. The most severe truncation generates what we will call the *conditional one particle entropy* (C1PE),

$$\Delta S_{1|s}=S_{1\text{ solute}}.$$ (29)

This is achieved by removing all integrals with a subscript greater than 1. If we include the two-body terms, then we have the *conditional two particle entropy* (C2PE),

$$\Delta S_{2|s}=S_{1\text{ solute}}-I_{2\text{ solute}}-S_{2\text{ liquid}}.$$ (30)

It is $\Delta S_{2|s}$ that this work is concerned with calculating.

*Step 4*. Instead of directly integrating numerically the terms in Eqs. (29) and (30), we can instead convert the integral into a sum which converges to the integral asymptotically in the limit of infinite data. We can then extrapolate toward that infinite limit by measuring the sum for finite quantities of data and fitting an appropriate extrapolating function. The estimators we use in this study are KNN estimators and are formulated as follows.

Inserting the explicit integral terms into Eq. (30) gives

$$\begin{array}{cc} \Delta S_{2|s} & =-R\rho \int g_{N}^{\left(1\right)}\left({𝐫}_{1}|s\right)\ln g_{N}^{\left(1\right)}\left({𝐫}_{1}|s\right)d{𝐫}_{1}\\ & -\frac{R\rho^{2}}{2}\int \int g_{N}^{\left(2\right)}\left({𝐫}_{1},{𝐫}_{2}|s\right)\ln\frac{g_{N}^{\left(2\right)}\left({𝐫}_{1},{𝐫}_{2}|s\right)}{g_{N}^{\left(1\right)}\left({𝐫}_{1}|s\right)g_{N}^{\left(1\right)}\left({𝐫}_{2}|s\right)}d{𝐫}_{1}d{𝐫}_{2}\\ & +\frac{R\rho^{2}}{2}\int \int g_{N}^{\left(2\right)}\left({𝐫}_{1},{𝐫}_{2}\right)\ln g_{N}^{\left(2\right)}\left({𝐫}_{1},{𝐫}_{2}\right)d{𝐫}_{1}d{𝐫}_{2}, \end{array}$$ (31)

we may separate out the denominators of the logarithm in the second term and then swap the coordinates **r**_1_ and **r**_2_ in one of those new integrals to give

$$\begin{array}{cc} \Delta S_{2|s} & =-R\rho \int g_{N}^{\left(1\right)}\left({𝐫}_{1}|s\right)\ln g_{N}^{\left(1\right)}\left({𝐫}_{1}|s\right)d{𝐫}_{1}\\ & -\frac{R\rho^{2}}{2}\int \int g_{N}^{\left(2\right)}\left({𝐫}_{1},{𝐫}_{2}|s\right)\ln g_{N}^{\left(2\right)}\left({𝐫}_{1},{𝐫}_{2}|s\right)d{𝐫}_{1}d{𝐫}_{2}\\ & +R\rho^{2}\int \int g_{N}^{\left(2\right)}\left({𝐫}_{1},{𝐫}_{2}|s\right)\ln g_{N}^{\left(1\right)}\left({𝐫}_{1}|s\right)d{𝐫}_{1}d{𝐫}_{2}\\ & +\frac{R\rho^{2}}{2}\int \int g_{N}^{\left(2\right)}\left({𝐫}_{1},{𝐫}_{2}\right)\ln g_{N}^{\left(2\right)}\left({𝐫}_{1},{𝐫}_{2}\right)d{𝐫}_{1}d{𝐫}_{2}, \end{array}$$ (32)

and it is then possible to integrate over **r**_2_ in the third term giving

$$\begin{array}{cc} \Delta S_{2|s} & =R\left(N-2\right)\rho \int g_{N}^{\left(1\right)}\left({𝐫}_{1}|s\right)\ln g_{N}^{\left(1\right)}\left({𝐫}_{1}|s\right)d{𝐫}_{1}\\ & -\frac{R\rho^{2}}{2}\int \int g_{N}^{\left(2\right)}\left({𝐫}_{1},{𝐫}_{2}|s\right)\ln g_{N}^{\left(2\right)}\left({𝐫}_{1},{𝐫}_{2}|s\right)d{𝐫}_{1}d{𝐫}_{2}\\ & +\frac{R\rho^{2}}{2}\int \int g_{N}^{\left(2\right)}\left({𝐫}_{1},{𝐫}_{2}\right)\ln g_{N}^{\left(2\right)}\left({𝐫}_{1},{𝐫}_{2}\right)d{𝐫}_{1}d{𝐫}_{2}. \end{array}$$ (33)

It is convenient to construct estimators to measure three distinct quantities

$$H_{1,s}=\rho \int g_{N}^{\left(1\right)}\left({𝐫}_{1}|s\right)\ln g_{N}^{\left(1\right)}\left({𝐫}_{1}|s\right)d{𝐫}_{1},$$ (34)

$$H_{2,s}=\rho^{2}\int \int g_{N}^{\left(2\right)}\left({𝐫}_{1},{𝐫}_{2}|s\right)\ln g_{N}^{\left(2\right)}\left({𝐫}_{1},{𝐫}_{2}|s\right)d{𝐫}_{1}d{𝐫}_{2},$$ (35)

$$H_{2,l}=\rho^{2}\int \int g_{N}^{\left(2\right)}\left({𝐫}_{1},{𝐫}_{2}\right)\ln g_{N}^{\left(2\right)}\left({𝐫}_{1},{𝐫}_{2}\right)d{𝐫}_{1}d{𝐫}_{2},$$ (36)

giving an expression for the conditional one- and two-particle entropies in terms of these estimators

$$\Delta S_{1|s}=RH_{1,s},$$ (37)

$$\Delta S_{2|s}=R\left(N-2\right)H_{1,s}+\frac{R}{2}\left(H_{2,l}-H_{2,s}\right).$$ (38)

In general, we may state the information entropy of a probability density function over *p* random variables as the *p*-dimensional integral

$$H\left[\rho \right]=-\int \rho \left({\left\{𝐱\right\}}_{p}\right)\ln\rho \left({\left\{𝐱\right\}}_{p}\right)d{\left\{𝐱\right\}}_{p}$$ (39)

as the expectation

$$H\left[\rho \right]=𝔼\left[-\ln\rho \left({\left\{𝐱\right\}}_{p}\right)\right].$$ (40)

However, in Eqs. (34)–(36), we have correlation functions in the logarithm rather than densities. We may then instead calculate

$$H\left[\rho \right]+\int \rho \left({\left\{𝐱\right\}}_{p}\right)\ln\rho d{\left\{𝐱\right\}}_{p}.$$ (41)

This quantity can then be approximated by a finite sum which is performed in Sec. II A.

### A. k-Nearest Neighbours (KNN) estimators

To efficiently calculate the integrals in Eqs. (29) and (30), we used the k-nearest neighbours method which has shown previous success in calculating first order solvation entropies,^10,13,23^ dihedral entropies in small molecules,^34^ and entropies of water in protein binding pockets.^14,22^ The general estimator for *N*_*p*_, *p*-dimensional objects across *F* frames of data is given by

$$H^{\left(k\right)}≅\frac{1}{N_{p}F}\sum_{i=1}^{N}\sum_{j=1}^{F}\ln\left(\frac{N_{p}\left(F-1\right)\pi^{p/2}d_{ij,k}^{p}}{\Gamma \left(\frac{p}{2}+1\right)}\right)-\psi \left(k\right),$$ (42)

where Γ(x) is the Euler gamma function, ψ(k) is the Euler digamma function, and the symbol ≅ denotes an asymptotic equivalence in the limit of an infinite number of frames *F*. This estimator was initially worked on for the first nearest neighbour by Leonenko^20^ and extended for the *k*th nearest neighbour by Singh^21^ and has been used by various studies.^10,13,14,19,34,35^ This estimator is a limiting case of the adaptive anisotropic elliptic kernel estimators.^36,37^

The estimator for an *H*_1_ term, given *F* sufficiently uncorrelated MD frames containing *N* neon atoms, is given by the expression

$$H_{1}^{\left(k\right)}≅\frac{1}{NF}\sum_{i=1}^{N}\sum_{j=1}^{F}\ln\left(\frac{4N\left(F-1\right)\pi d_{ij,k}^{3}}{3V}\right)-\psi \left(k\right),$$ (43)

which is used in Refs. 13 and 14. The next order expression is then

$$H_{2}^{\left(k\right)}≅\frac{2}{N\left(N-1\right)F}\times \sum_{i_{1}=1}^{N}\sum_{i_{2}>i_{1}}^{N}\sum_{j=1}^{F}\ln\left(\frac{N\left(N-1\right)\left(F-1\right)\pi^{3}d_{i_{1}i_{2}j,k}^{6}}{6V^{2}}\right)-\psi \left(k\right).$$ (44)

In previous work, Eqs. (43) and (44) have been shown to fit a power law for increasing values of *F*^13,14,19^

$$H_{k}\left(F\right)=a_{k}F^{b_{k}}+H_{\infty }$$ (45)

with *a*_*k*_ and *b*_*k*_ some constants for the *k*th neighbour selected, and $H_{\infty }$ the asymptotic value of the entropy. *b*_*k*_ is a negative constant such that

$$\lim_{F\to \infty }H_{k}\left(F\right)=H_{\infty }.$$ (46)

To extract this value, a power law can be fitted to *H*(*F*).

## III. SIMULATION DETAILS

FEP and IFST were used to calculate the free energy change associated with adding a fixed Lennard-Jones particle to the centre of a neon simulation box in the canonical ensemble. The IFST free energy with only $\Delta S_{1|s}$ and with both $\Delta S_{1|s}$ and $\Delta S_{2|s}$ were calculated. All of the simulation data used in this study came from the same forcefield and MD simulations were all carried out in NAMD.^38^ Thus, the resulting free energies are directly comparable.

### A. Systems setup

The neon MD parameters were taken from the CHARMM27 forcefield.^39^ There are no electrostatic interactions for this solvent-solute system, so all potential energy terms are in the form of a Lennard-Jones potential

$$V_{LJ}\left(r_{ij}\right)=\sqrt{\varepsilon_{i}\varepsilon_{j}}\left({\left(\frac{R_{i}+R_{j}}{2\left|r_{ij}\right|}\right)}^{12}-2{\left(\frac{R_{i}+R_{j}}{2\left|r_{ij}\right|}\right)}^{6}\right),$$ (47)

where *r*_*ij*_ is the distance between atoms *i* and *j*, and *R*_*k*_ is the radius of minimum potential energy for atom *k*, $R_{k}=2^{1/6}\sigma_{k}$. 4 different solutes were taken which had varying Lennard-Jones $\varepsilon_{s}$ parameters as shown in Table I. All solutes had the same *R*_*k*_ parameter as the neon solvent. Each solute was initially solvated with 900 neon atoms in a cubic box of edge length 27.5Å. Where reduced units are given for reference they are calculated as the temperature $T^{*}=k_{B}T/\varepsilon$, pressure $P^{*}=p\sigma^{3}/\varepsilon$, length $L^{*}=L/\sigma$, and number density $\rho^{*}=\rho \sigma^{3}$.

**TABLE I.** Forcefield parameters used for each of the solute atoms where $\varepsilon_{n}$ is the value for bulk neon.

Solute name	$\varepsilon_{s}$ [kcal/mol]	*R*_*k*_ [Å]	*L*	*ρ* [$\text{Ne}{\text{Å}}^{-3}$]	*T* (K)
SOL1	0.215	3.06	27.3760	0.043 82	25
SOL2	0.430	3.06	27.3710	0.043 84	25
SOL3	0.645	3.06	27.3707	0.043 84	25
SOL4	0.860	3.06	27.3685	0.043 85	25

Solute name	$\varepsilon_{s}/\varepsilon_{n}$	$\sigma_{s}$ [Å]	$L^{*}$	$\rho^{*}$	$T^{*}$
SOL1	2.5	2.73	10.0420	0.8887	0.578
SOL2	5.0	2.73	10.0401	0.8892	0.578
SOL3	7.5	2.73	10.0400	0.8893	0.578
SOL4	10.0	2.73	10.0392	0.8895	0.578

For the 12-6 LJ fluid, the triple point density of both the liquid $\rho_{tl}^{*}$ and solid $\rho_{ts}^{*}$ phases have been measured by previous studies to be in the ranges $\rho_{tl}^{*}=0.818\text{–}0.864$ and $\rho_{ts}^{*}=0.96\text{–}0.978$, and the triple point has approximate temperature and pressure $T^{*}=0.661$ and $p^{*}=0.0018$, respectively.^40^ Thus, the simulated neon was in the liquid phase.

### B. IFST

#### 1. IFST equilibration

16 replicate equilibrations per solute type were performed in the NpT ensemble for 4 ns to find the natural densities of the simulations cells with each solute present. The resulting edge lengths are shown in Table I.

A 2 ns equilibration was then performed for each replicate in the NVT ensemble at *T* = 25 K ($T^{*}=0.578$) and 1 atm ($p^{*}=0.003 44$) using the Langevin temperature control. An MD timestep of 2.0 fs was used. Electrostatic interactions were turned off as they were not present in the forcefield. van der Waals interactions were removed for separations over 10.5Å, and switching was used between 9.5Å and 10.5Å. The simulations took place with cubic periodic boundary conditions. This process was repeated for each of the 4 solutes and one bulk system with no solute.

#### 2. IFST production

The production simulations were carried out in the NVT ensemble for 60 ns at 25 K ($T^{*}=0.578$) with the same MD parameters as the NVT equilibration. NAMD produces a trajectory (dcd) file, which is a set of system coordinate snapshots. A trajectory frame was saved every 500 fs such that neighbouring frames in the dcd file were not too strongly correlated; this is important for the KNN estimator when used later to extract entropies as commented in the previous work by Huggins.^13^ The energies $\Delta U={\bar{U}}_{solute}-{\bar{U}}_{bulk}$ were calculated by taking the average energy across all 16 repeats of the 60 ns production simulation, and these are shown in Table II along with the standard deviation across 16 repeats, $\sigma_{\Delta U}$.

**TABLE II.** Average free energy results for each solute. $\sigma_{\Delta U}$ is the standard deviation of the 16 energy results for each solute. $\sigma_{FEP}$ is the standard deviation across all 16 repeats of the FEP free energy change $\Delta A_{FEP}$. $\sigma_{1|s}$ and $\sigma_{2|s}$ are the standard deviations across all 16 repeats of the IFST free energy changes $\Delta A_{1|s}$ and $\Delta A_{2|s}$, respectively. All units are in kcal/mol.

	$\Delta A_{FEP}$	ΔU	$\Delta S_{1|s}$	$\Delta S_{2|s}$	$\Delta A_{1|s}$	$\Delta A_{2|s}$
SOL1	1.036	−1.717	−0.541	$<10^{-4}$	1.176	1.176
SOL2	1.820	−2.561	−0.589	$<10^{-4}$	1.972	1.972
SOL3	2.438	−3.257	−0.694	$<10^{-4}$	2.563	2.563
SOL4	2.975	−3.839	−0.773	$<10^{-4}$	3.067	3.067

	$\sigma_{FEP}$	$\sigma_{\Delta U}$	$\sigma_{\Delta S_{1|s}}$	$\sigma_{\Delta S_{2|s}}$	$\sigma_{1|s}$	$\sigma_{2|s}$
SOL1	0.004 95	0.0279	0.052	$<10^{-4}$	0.062	0.062
SOL2	0.003 82	0.0281	0.131	$<10^{-4}$	0.126	0.126
SOL3	0.005 85	0.0238	0.088	$<10^{-4}$	0.100	0.100
SOL4	0.006 24	0.0155	0.061	$<10^{-4}$	0.064	0.064

#### 3. IFST free energy calculations

To calculate the change in free energy for IFST simulations, we need to calculate each component on the right hand side of

$$\Delta A_{1|s}=\Delta U-T\Delta S_{1|s}$$ (48)

and

$$\Delta A_{2|s}=\Delta U-T\Delta S_{2|s}.$$ (49)

ΔU was calculated using Eq. (2), and therefore the quantities $\Delta S_{1|s}$ and $\Delta S_{2|s}$ must be calculated using Eqs. (37) and (38). This then requires collecting a set of nearest neighbour distances and nearest pair distances from the respective MD data for the entropy estimators.

### C. K-Dimensional (K-D) trees

Previous studies using IFST or GIST have used a grid of voxels or cut-cell approach to find nearest neighbour distances.^13,14^ For the pair nearest neighbour distances required for Eq. (44), this method was not practical. In order to increase the efficiency and allow the calculation of the pair terms, a K-Dimensional (K-D) tree method was used. This is covered in more detail in the supplementary material.

### D. Free energy perturbation (FEP)

#### 1. FEP protocol

The FEP simulation measures the change in free energy of removing a fixed solute from a box of neon. This simulation is parameterized by a variable λ such that when λ=0, the solute is fully present and when λ=1, the solute is fully annihilated.

**FIG. 2.**
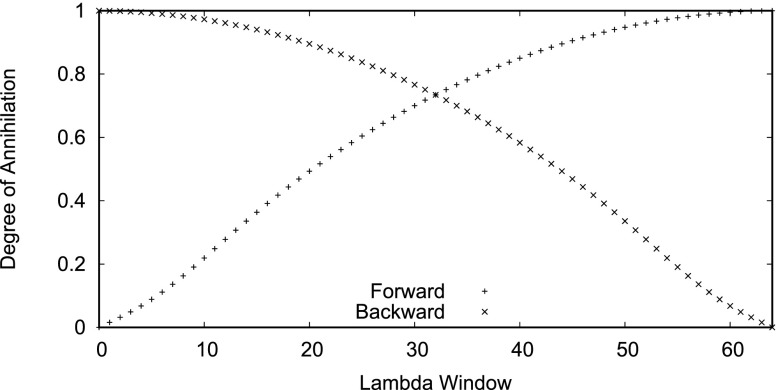
The lambda schedule for the forward and backward FEP simulations across the 64 lambda windows as generated by Eqs. (50) and (51).

In this study, each forward and backward FEP simulation had *N* = 64 “*λ*-windows,” and each window has a different value of lambda which is labeled as *degree of annihilation* in the schedule displayed in Fig. 2. For the *n*th window out of a total of *N* windows, the value of *λ* in that window was generated with the expressions

$$\lambda_{f}\left(n\right)=\frac{1}{N}\sum_{k=1}^{N}1-{\left(1-\frac{n}{N}\right)}^{\max\left(k-N+n,1\right)},$$ (50)

$$\lambda_{b}\left(n\right)=\frac{1}{N}\sum_{k=1}^{N}1-{\left(\frac{n}{N}\right)}^{\max\left(k-n,1\right)},$$ (51)

which satisfy $\lambda_{f}\left(0\right)=0$,$\lambda_{f}\left(N\right)=1$,$\lambda_{b}\left(0\right)=1$, and $\lambda_{b}\left(N\right)=0$, where $\lambda_{f}\left(n\right)$ and $\lambda_{b}\left(n\right)$ are the forward and backward schedules, respectively. These curves were picked to sample the endpoints of the FEP simulation more heavily to “avoid the end point catastrophe.”^41^ The endpoints are when the solute is close to full annihilation in the forward simulation (n≈N) or when the solute is just being created in the backward simulation (n≈0). A van der Waals soft core potential parameter of 5.0 was used. One energy reading was stored every 100 steps which equates to every 0.2 ps.

#### 2. FEP equilibration

The end points of the NVT equilibrations for the IFST simulations were used as starting points for the FEP simulations. Each solute had 16 replicates. Each simulation underwent a further 0.2 ns equilibration at *T* = 25 K ($T^{*}=0.578$) from this point to adjust to their individual λ values.

#### 3. FEP simulation

For each *λ*-window, 0.469 ns of simulation was performed in the NVT ensemble at *T* = 25 K ($T^{*}=0.578$). Then, considering the 64 windows in both directions the overall time was 60.1 ns, which is comparable to the 60 ns used for the IFST production run.

#### 4. FEP calculations

The final change in the free energy from the FEP simulations is found by summing the changes in free energy between each pair of neighbouring *λ* windows. Then, the forward change is

$$\Delta A_{f,FEP}=\sum_{n=0}^{63}\Delta A_{\lambda \left(n\right),\lambda \left(n+1\right)}$$ (52)

and the backward change is

$$\Delta A_{b,FEP}=\sum_{n=1}^{64}\Delta A_{\lambda \left(n-1\right),\lambda \left(n\right)}.$$ (53)

To calculate the changes in free energy between the *λ*-windows, the Bennett Acceptance Ratio (BAR) method was used,^42^ which is included in the ParseFEP Plugin^43^ for Visual Molecular Dynamics (VMD)^44^ in which all FEP results for this study were calculated. The BAR estimator provides a calculated statistical error, and these errors were less than 0.007 kcal/mol for all of the 64 simulations.

## IV. RESULTS AND DISCUSSION

### A. Results

**FIG. 3.**
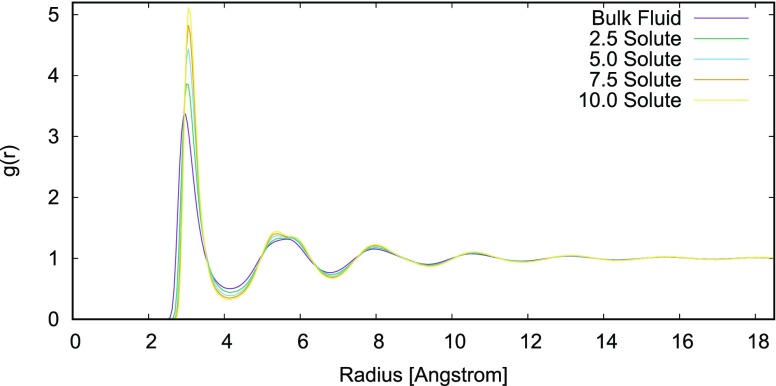
The radial distribution functions for the solute to all fluid atoms. The fluid-fluid RDF is also plotted. For stronger central potentials, the peaks get higher and the troughs get deeper along with a consistent distortion in the second solvation shell.

Fig. 3 shows the solute-fluid radial distribution functions for all solutes and bulk neon.

**FIG. 4.**
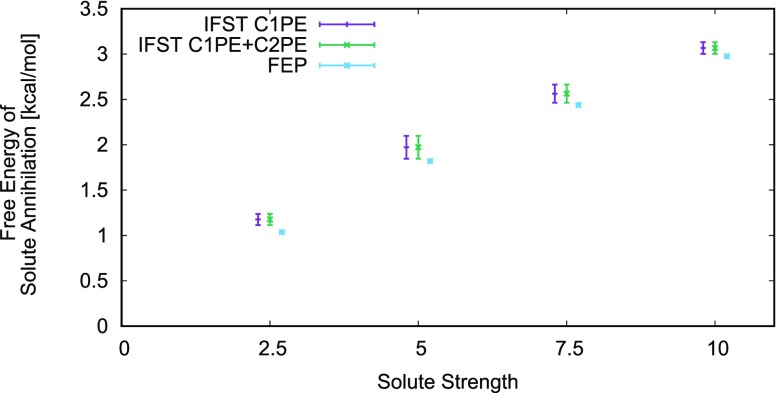
The free energy estimates for the two IFST approximations and the FEP result at $\varepsilon_{s}$ values of $2.5\varepsilon_{n},5.0\varepsilon_{n},7.5\varepsilon_{n}$, and $10.0\varepsilon_{n}$. Different levels of theory have been spaced for clarity. C1PE is the conditional one-particle entropy correction. C2PE is the conditional two-particle entropy correction. The error bars are the spread in the 16 repeats of each result.

Fig. 4 shows a comparison of the free energy estimates from both levels of the IFST results and from FEP.

Table II shows the comparison of the average free energies from FEP and IFST calculations, and the IFST calculations show the conditional one-particle and conditional two-particle entropy values.

**FIG. 5.**
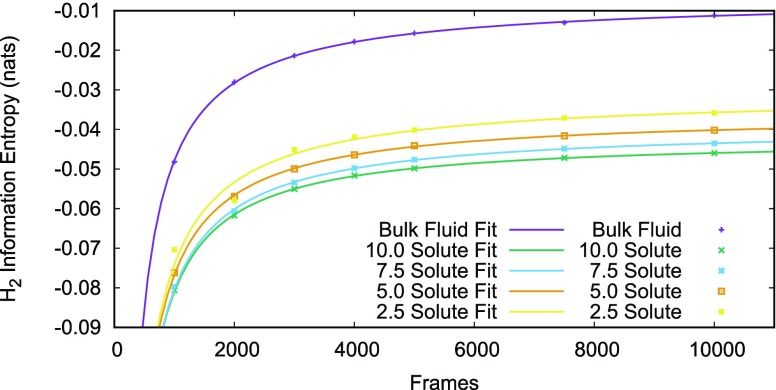
A plot demonstrating how the asymptotic values for the *H*_*s*,2_ and *H*_*l*,2_ estimators were extracted. A power law is fitted to the three data points for each solute. The constant in the limit of infinite frames is used as in Eq. (45).

The IFST free energies in Table II were calculated by Eqs. (48) and (49), the entropy terms were calculated by extrapolating Eq. (45) for the conditional one-particle entropies every 1000 frames from 1000 to 12 000, and Eq. (45) for conditional two-particle entropies, 16 repeats were taken at intervals of 1000, 2000, and 3000 frames and 2 repeats were extended to 1000, 2000, 3000, 4000, 5000, 7500, and 10 000 frames. Fig. 5 shows an example of the conditional two-particle entropy extrapolation for all solutes. The extrapolation routine used was from the gnuplot software, when fitting it weighted data points by the standard deviation of the 16 repeats of that point. For the conditional two-particle entropy fitting, where no standard deviation was available it was estimated to be 10/*F* kcal/mol, where *F* is the number of frames for that data point.

### B. Discussion

Fig. 3 shows the radial distribution functions (RDFs) from the central solute to all solvent atoms. The troughs become deeper and the peaks become higher for increased solute potential $\varepsilon_{s}$ parameter. Also plotted is the RDF for the bulk solvent, which has the shallowest troughs and lowest peaks. There is a slight distortion in the second solvation shell, which becomes more pronounced with higher $\varepsilon_{s}$. This is likely to be a gentle crowding effect as the solvent molecules in the first solvation shell draw close to the solute, and the closest locations for second shell atoms correspond to the pits in the already tightly packed first solvation shell. The magnitude of the solvent-solvent correlation entropy was expected to be small in the system of monatomic species used in this study. It has been shown that in the Lennard-Jones system, the primary source of the solvent structure comes from packing,^45^ and this will correspond to an entropy associated with the volume exclusion of the central solute which is captured fully by the C1PE term. For more complex solvents, namely water, evidence already exists for the solvent-solvent correlations.^46,47^

Fig. 4 shows the calculations of the free energy of solute annihilation from all three methods of evaluation with the standard deviations of 16 repeats used as error bars. The average values of these estimates are displayed in Table II along with the standard deviations of the 16 repeats. The average values for all methods are in good agreement. The IFST results including the conditional two-particle correction have the same averages and standard deviations of the first order IFST. This demonstrates that the C2PE has no clear contribution to the free energy of this system. The FEP results have the lowest standard deviation of all results even though a comparable length of MD run was used. The increased uncertainty associated with the IFST results likely arises from fitting power laws to extract the asymptotic entropy. The standard deviation in the free energy is at least 3 times greater than that of the MD energies used, which indicates the added uncertainty is from the entropy. This could potentially be remedied by taking more data points during the extrapolation process at the expense of data processing time. Both the average IFST results are within 0.16 kcal/mol of the FEP results. These results indicate that it is possible to reconstruct a fairly accurate measurement of the free energy change of this kind of process by only using the start and end points of an equivalent FEP path. The results indicate that the entropy term contributes relatively less to the free energy of solute annihilation for solutes with larger *ε*.

## V. CONCLUSIONS

The translational entropy associated with the solute-solvent correlation and the solvent-solvent correlation can be used to evaluate the free energy of solvation in the IFST framework for a system of neon atoms solvating a fixed central Lennard-Jones potential. Both IFST estimates reproduce the estimate by an equivalent FEP simulation to within around 0.16 kcal/mol and appear to consistently overestimate slightly. The IFST method has an advantage over FEP and can give a free energy estimate without having to define a path between the two systems. However, the accuracy associated with FEP estimates is greater, so it is a better tool for computation. We conclude that IFST in its current state is the better tool for physical interpretation as it avoids the non-physical lambda states utilized by FEP. We note that FEP is an already well-developed method, and IFST may yet have future improvements to increase its optimization. The conditional two-particle entropy term did not contribute a noticeable change in free energy for this system for any of the strengths of the solute potential tested.

The IFST framework can give the spatial contributions of configurational entropy when analysed with voxel based methods (as shown in Fig. 3 of the supplementary material). This allows areas of interest around the solute to be highlighted. Although the two-particle terms in the MIE do not appear to be significant in this simple system, they may be significant in a system with a liquid water solvent with hydrogen bonding and charges. The methods used in this work may be extended to such a system.

If the solvent-solvent interactions become very strong in a particular solvation process, it may be necessary to calculate the *conditional three-particle entropy*

$$\Delta S_{3|s}=S_{1\text{ solute}}-I_{2\text{ solute}}+I_{3\text{ solute}}-S_{2\text{ liquid}}+I_{3\text{ liquid}}$$ (54)

and the methodology used in this work could be extended to such a calculation.

## SUPPLEMENTARY MATERIAL

See supplementary material for a description of the implementation of K-D trees in this work, and for a spatial illustration of the C2PE contributions around a fixed solute. The data and codes used for calculations in this work will be available at https://doi.org/10.17863/CAM.9335
